# Development of the Japanese version of the perceived time poverty scale

**DOI:** 10.1371/journal.pone.0320807

**Published:** 2025-04-01

**Authors:** Takeshi Miura, Koji Hara, Azusa Arimoto, Masato Kaneko, Sayuri Shiraishi, Shingo Matsumura, Shuichi Ito, Kentaro Kurasawa, Yohei Matsuzaki, Makoto Kuroki

**Affiliations:** 1 Department of Health Data Science, Yokohama City University, Yokohama, Kanagawa, Japan; 2 Nursing Course, School of Medicine, Yokohama City University, Yokohama, Kanagawa, Japan; 3 School of Economics and Business Administration, Yokohama City University, Yokohama, Kanagawa, Japan; 4 Department of Pediatrics, Graduate School of Medicine, Yokohama City University, Yokohama, Kanagawa, Japan; 5 Department of Obstetrics & Gynecology, Women’s Health Yokohama City University Hospital, Yokohama, Kanagawa, Japan; Chiba Daigaku, JAPAN

## Abstract

**Background:**

Employed households experience time poverty, which refers to feeling overwhelmed because of the struggle to balance work and life. Time poverty is subjectively perceived as a lack of personal free time. In Japan, long working hours and societal expectations regarding the division of work and family roles may influence the perception of time poverty. This issue is of significant concern, as it can impact individuals’ rest time and work productivity. However, there is currently no standardized measurement method to assess time poverty appropriately in the Japanese context. The lack of such a method challenges establishing a foundation for developing effective support strategies. Given this background, this study aimed to quantify time poverty among employed households by developing a Japanese version of the Perceived Time Poverty Scale and examining its reliability and validity.

**Methods:**

In developing the Japanese version of the Perceived Time Poverty Scale, cultural adaptations were made in addition to the standard translation and back-translation procedures. Through discussions with researchers and translation experts, terms with differing scopes of interpretation in the Japanese context were revised, and expressions were adjusted to reflect the intended concepts better. The data for this study were collected through Wave 2 of the longitudinal survey, the Hama Study, conducted over a five-year period from 2022 to 2027. This survey randomly selected 10,000 employed households residing in Yokohama, Japan. Participants completed the Japanese version of the Perceived Time Poverty Scale developed in this study, along with the well-being scale, the Kessler Screening Scale for Psychological Distress, and the Japanese Short-Form UCLA Loneliness Scale. Exploratory and confirmatory factor analyses were conducted to evaluate the scale structure. Internal consistency was assessed using Cronbach’s alpha and McDonald’s omega coefficients. Furthermore, correlations between the Japanese version of the Perceived Time Poverty Scale and the other scales were examined to evaluate the structural validity of the scale.

**Results:**

Data from 1,979 respondents who participated in the Wave 2 online survey were analyzed. The scale demonstrated high reliability, with a Cronbach’s alpha coefficient 0.90 (95% CI: 0.89–0.91). Exploratory factor analysis confirmed a single-factor structure and confirmatory factor analysis supported this structure with fit indexes (CFI =  0.957, TLI =  0.929, RMSEA =  0.136, SRMR =  0.035). Perceived time poverty was negatively correlated with sleep time and leisure time, and positively correlated with childcare time. Furthermore, perceived time poverty showed significant correlations with well-being, psychological distress, social isolation, and job satisfaction, confirming the validity of the developed scale.

**Conclusion:**

The Japanese version of the Perceived Time Poverty Scale is a reliable tool with a certain degree of validity for assessing time poverty in Japan. This scale enables individuals and households to recognize time poverty as a modern form of poverty. Furthermore, businesses and local governments can utilize it as an indicator in practical settings, such as improving work environments, implementing childcare support programs, and promoting community health. Future longitudinal studies are needed to further validate the scale, including addressing issues related to model fit.

## Introduction

Employed households in Japan and worldwide have increasingly reported experiencing a sense of being excessively busy and lacking sufficient time [1]. This phenomenon has been recognized as a new form of poverty embedded in the lifestyle patterns of modern society [2,3]. Surveys suggest that many employed households deal with a growing sense of time scarcity, as individuals must juggle multiple responsibilities, such as work, household chores, childcare, and caregiving. Consequently, leisure time and sleep are becoming limited [4–6].

Several key factors contribute to time poverty in employed households. The rise of dual-income couples is a significant element, as both partners work longer hours, increasing commuting times. In addition, the constant need to stay connected through online channels has increased work-related tasks and frequent interruptions during personal time. Moreover, the pressure of task-oriented productivity has resulted in higher expectations for efficiency within shorter timeframes. Psychologically, individuals prioritize monetary gain over personal time, creating an imbalance between work, personal, and family life [7,8]. This imbalance can ultimately lead to a decline in overall well-being.

The impact of time poverty on the health and well-being of employed households has been extensively documented. Several studies have shown that time poverty is associated with issues such as obesity [9], mental stress [10], overall well-being [11], and decline in self-assessment and mental health [12]. This highlights the urgent need for a quantitative assessment of time poverty among employed households. This assessment would allow for a better understanding of the individual and regional characteristics contributing to time poverty and could aid policymakers in developing effective measures to address the issue. This could, in turn, improve productivity, enhance well-being, and contribute to balanced work–life environments.

Time poverty is a state in which individuals cannot secure the minimum necessary time for basic living needs owing to work demands [13]. However, inconsistent definitions across research fields and varied concepts and measurement methods of time poverty among researchers make it challenging to reach a unified understanding of this concept [14]. For instance, some studies define time poverty as a chronic sense of having too many tasks and not enough time to complete them [1]. By contrast, others refer to it as insufficient discretionary time available for personal use [15]. Measurement methods have similarly varied, with some focusing on the available time after deducting paid working hours [16] and others emphasizing available leisure time [17].

To address these conceptual and measurement challenges, Zheng et al. [14] developed the Perceived Time Poverty Scale (PTPS), a subjective measure designed to quantify individuals’ perceptions of having insufficient discretionary time. This six-item scale demonstrated strong reliability and validity across various socioeconomic and cultural backgrounds [14]. The PTPS offers a consistent and comprehensive method of measuring time poverty, which significantly impacts well-being and productivity. The scale’s development marks a major advancement in understanding the effects of time poverty on individuals and society.

Despite the global significance of time poverty, no validated scale has been developed to assess it within the Japanese population. This presents a critical gap in research and policy development. Therefore, this study aimed to create a Japanese version of the PTPS and to evaluate its reliability and validity among employed households in Japan. Previous studies on time poverty have primarily focused on specific professions, such as school teachers [18], while comprehensive examinations targeting employed households in general have been limited. In addition, earlier assessments frequently relied on time-consuming recall methods, significantly burdening respondents [19]. By contrast, the concise six-item structure of the PTPS offers a more practical and comprehensive tool with minimal burden on respondents.

## Materials and methods

### Development of the Japanese version of the perceived time poverty scale

We translated the PTPS into Japanese through the following process in accordance with the guidelines for scale translation [20]. Prior to the translation, permission was obtained from the lead author of the original study, Xingshan Zheng on September 13, 2023, after explaining our objectives and translation process.

The scale was independently translated into Japanese by three translators who were native Japanese speakers. This included two researchers familiar with the scale’s purpose (specializing in medicine and business) and one professional translator from a translation company. The translated versions were then discussed among five experts in medicine, management, and labor economics—co-authors of this study who share a common interest in addressing the global issue of declining birth rates—regarding their integration into a draft Japanese version. This draft underwent back-translation by a professional translation agency, and the back-translated version was reviewed and discussed with Xingshan Zheng, leading to the finalization of the Japanese version. This finalized version was further reviewed by employed individuals, both men and women, to ensure clarity in the instructions, response format, and item wording.

While translating the scale into Japanese, “private” was initially used in the phrase “I planned in my private life.” However, it was revised to “personal,” as “private” could imply leisure activities, such as entertainment, travel, or exercise. Additionally, during the back-translation, the Japanese term initially translated as “like” was reviewed, because the original English version used “love” to emphasize the importance of personal activities. Researchers explained that the item was intended to capture the discretionary time individuals spend on activities they are passionate about. However, the distinction between “love” and “like” was not considered likely to significantly impact the perception of having time for such activities. Based on the Japanese context, the term “like” was ultimately deemed more appropriate and was adopted.

### Survey methods

This study analyzed data from the prospective cohort study titled Questionnaire Survey of Married and Child-Rearing Households (Hama Study), conducted in Yokohama, focusing on time poverty among households balancing work and child-rearing [21]. This longitudinal survey aims to track lifestyle and health changes over a five-year period from 2022 to 2027. This cohort was a large-scale survey conducted in collaboration between Yokohama City University and the City of Yokohama, making it suitable for the objectives of this study, which targets employed households. Consequently, it was incorporated into Wave 2 of the longitudinal study. Furthermore, this cohort was selected because it allows for longitudinal analysis until 2027, enabling an in-depth examination of changes in time poverty and its contributing factors over time. Yokohama is one of Japan’s major cities, characterized by a diverse population and a high concentration of employed households. The insights gained from this region are expected to provide valuable considerations for understanding the challenges faced by employed households in balancing work and family life within an urban environment. The survey randomly selected 10,000 married couples residing in Yokohama, among whom the wives were born between April 2, 1983, and April 1, 2001, making them 20–39 years of age in 2022. Families without children or with children under 6 years old (preschoolers) were included in the study. Exclusion criteria included couples for whom either spouse was not living in Yokohama at the time of the survey. Additionally, participants who did not provide consent or gave incomplete responses were excluded from the analysis.

The first survey results were published in 2023 by the Yokohama City Children and Youth Bureau and Yokohama City University [21–23]. The PTPS was integrated into the second survey, conducted from January to February 2024, which covered a broad range of work and family life aspects. Each household was sent two identical survey forms, one for the wife and one for the husband, with instructions for them to respond independently. Participants could return the completed survey forms via the enclosed return envelope or submit their responses through an online survey form. A written explanation of the study accompanied the survey, and informed consent was obtained through a checkbox on the form, which participants marked to confirm their agreement to participate. Only surveys with confirmed consent were included in the analysis. The survey instructions explained voluntary participation, the purpose of the study, and how the research findings would be utilized. Additionally, a dedicated website was established to disseminate information about the study, providing participants with updates and fostering transparency. The survey data did not include personally identifiable information, such as names, to ensure anonymity and confidentiality. The collected data were securely stored and accessible only to authorized research personnel.

### Survey items

The questionnaire for the second survey consisted of a total of 35 items, including 6 items on demographic characteristics, 6 items on the PTPS, 6 items on the average daily schedule, 1 item on well-being, 6 items on the Kessler Screening Scale for Psychological Distress (K6), and 10 items on the Japanese version of the Short-Form UCLA Loneliness Scale (version 3). The questionnaire included scales previously validated for reliability and validity in prior research. The average daily schedule was selected to examine the relationship between quantitatively assessed actual time experienced by the participants and subjective time poverty. Furthermore, these data clarify the differences between the objective aspects of time use and subjective perceptions, contributing to a comprehensive understanding of time poverty. The well-being, K6, and UCLA scales were selected based on the hypothesis that time poverty affects individuals psychologically.

### Demographic characteristics

The following demographic characteristics were obtained: sex, age, employment status, number of children, household income, and educational background. Age was calculated based on the Basic Resident Register in Yokohama City as of January 1, 2024. Employment status was categorized as “employed” if respondents reported having a job, including full-time, part-time, self-employed, or being on childcare leave. Those who reported not currently working, including students and homemakers, were classified as “unemployed.” The respondent’s number of children was categorized into five groups: 0, 1, 2, 3, and 4 or more. Educational background was classified into four categories: high school, technical school, bachelor’s degree, and graduate degree. Household income was classified into four categories: less than 6 million yen, 6 million to less than 9 million yen, 9 million to less than 15 million yen, and 15 million yen or more.

### Japanese version of the perceived time poverty scale

The scale items assess the extent to which respondents feel they lack time for various activities: “I often feel that I do not have time for socializing,” “I often feel that I do not have time for leisure activities,” “I often feel that I do not have time to travel,” “I often feel that I do not have time to exercise,” “I often feel that I do not have time to do the things I like,” and “I often feel that I do not have time to do things that I have planned in my personal life.” Responses were rated on a seven-point Likert scale (1 =  strongly disagree; 7 =  strongly agree). The total score was calculated by summing the item scores, with higher scores indicating a higher (likelihood of) time poverty. The scale’s Likert format allows for nuanced responses, capturing varying levels of perceived time poverty across different aspects of daily life.

### Psychological distress

Psychological distress was measured using the K6, developed by Kessler et al. [24] and validated in Japanese [25,26]. The six items ask respondents how often they experienced the following feelings during the past 30 days: “nervous,” “hopeless,” “restless or fidgety,” “so depressed that nothing could cheer you up,” “that everything was an effort,” and “worthless.” Responses were rated on a five-point Likert scale (1 =  never; 5 =  always). Total scores ranged from 6 to 30, with higher scores indicating higher levels of psychological distress. The K6 has been widely used in clinical and research settings to assess general psychological distress and has shown good reliability and validity in the Japanese population.

### Loneliness

Loneliness was measured using the Japanese version of the 10-item Short-Form UCLA Loneliness Scale (version 3) was used, developed by Russell [27] and validated by Arimoto and Tadaka [28]. The scale comprises the following items: “Do you often feel that you lack companionship?” “Do you often feel that you have a lot in common with the people around you?” “Do you feel that there are people who are close to you?” “Do you often feel left out?” “Do you often feel that there is no one who really knows you well?” “Do you often feel isolated from others?” “Do you feel that there are people who really understand you?” “Do you often feel that you are not part of a group with the people around you?” “Do you feel that there is no one to talk to?” and “Do you feel that there are people you can rely on?” Responses were rated on a four-point Likert scale (1 =  never; 4 =  always). Total scores ranged from 10 to 40, with higher scores indicating greater loneliness. The scale has been widely used to measure subjective feelings of loneliness and social isolation and has demonstrated good reliability and validity in the Japanese population. A higher score reflects a stronger sense of social isolation and emotional loneliness, whereas a lower score indicates greater connectedness with others.

#### Average daily schedule.

Respondents reported the average time spent on various daily activities, such as sleep, work, commuting, childcare, housework, and leisure, on weekdays.

#### Well-being.

Well-being was rated on a scale from 0 (very unhappy) to 10 (very happy). This single-item measure allowed the participants to self-assess their overall happiness and life satisfaction.

#### Job satisfaction.

Job satisfaction was assessed on a scale from 0 (very dissatisfied) to 10 (very satisfied). This measure captured the participants’ overall contentment with their current job situation.

### Statistical analysis

This study analyzed data from 1,979 participants who responded online to the Hama study. The participants were randomly divided into two subsets: data of 989 participants were used for exploratory factor analysis (EFA), and data of 990 participants were used for confirmatory factor analysis (CFA). All statistical analyses were conducted using R version 4.3.3. In all cases, p < .05 was considered statistically significant, ensuring that any differences observed were unlikely to have occurred by chance. Missing values were excluded using the listwise deletion method.

#### Item analysis.

Guttman split-half reliability (G-P) analysis was conducted to evaluate each item’s discrimination power. This method enables comparison of the upper and lower groups to assess whether the scale items have appropriate discrimination power. The total scores for the scale were calculated, and participants were divided into two groups based on their scores: the upper group (top 25%) and the lower group (bottom 25%). A t-test was then performed to determine whether significant differences existed between these two groups for each item. This method helped identify items that effectively differentiated between high and low scorers on the scale. In addition, an item-total (I-T) analysis was used to examine the correlation between each item and the total scale score and assess whether each item contributed meaningfully to the overall scale.

#### Exploratory factor analysis.

An EFA was conducted to explore the underlying factor structure of the PTPS. The number of factors to retain was determined by examining a scree plot and the parallel analysis results. This helped identify the most appropriate number of factors based on the data. A maximum likelihood estimation with varimax rotation was employed. This method is commonly used to ensure that the factors are independent and that the loadings of items on each factor are maximized. This process helped identify the structure of the scale and how items were grouped to form meaningful subscales.

#### Confirmatory factor analysis.

Subsequently, a CFA was conducted to test the model fit and factor loadings of the identified structure and confirm the validity of the factor structure suggested by the EFA using a different subset of data. Maximum likelihood estimation was employed, with varimax rotation applied. Model fit was evaluated using the chi-square to degrees of freedom ratio (χ²/df), comparative fit index (CFI), Tucker–Lewis index (TLI), root mean square error of approximation (RMSEA), and standardized root mean square residual (SRMR). These indexes collectively assess how well the proposed model fits the observed data.

χ^2^/df closer to 0 indicates a better fit, but values below 6 are also considered acceptable. CFI values of 0.95 or higher indicate a good fit, while values up to 0.90 are considered acceptable. Values greater than 0.90 for TLI, which accounts for model complexity, indicate a good fit. RMSEA values closer to 0 indicate a better fit, with values below 0.05 considered excellent and values up to 0.08 deemed acceptable. Additionally, it is recommended to report the 90% confidence interval (CI) for RMSEA. SRMR measures the discrepancies between predicted and observed data, with values below 0.08 suggesting an acceptable fit [29].

#### Reliability.

The reliability of the PTPS was evaluated using internal consistency measures, namely Cronbach’s alpha and McDonald’s omega. Cronbach’s alpha was used to measure the overall consistency of the scale, with values <  0.70 indicating acceptable reliability. McDonald’s omega provided a complementary measure of reliability, particularly for cases where the assumptions underlying Cronbach’s alpha were not fully met. Both indexes provided insights into the scale’s reliability when used with similar populations.

#### Validity.

To assess the validity of the PTPS, the correlations between the total PTPS score and the other measured dimensions (daily schedule, well-being, psychological distress, loneliness, and job satisfaction) were analyzed. We hypothesized that PTPS scores would be correlated with these variables, as they all theoretically related to time poverty. Pearson correlation coefficients were calculated to examine the strength and direction of these relationships and provide evidence for the convergent validity of the PTPS.

### Ethical considerations

Participants were provided with written information explaining the study objectives when completing the questionnaire, ensuring they had the opportunity to review the research details. Informed consent was obtained through a checkbox on the form, which participants marked to confirm their agreement to participate. Participation in the study was entirely voluntary, and participants were assured of their right to withdraw consent at any time without any consequences. No incentives or compensation were provided for participation. The questionnaire did not include any personally identifiable information, such as names or addresses, and all necessary measures were taken to ensure that individuals could not be identified from their responses, ensuring participants’ sense of security and confidentiality.

Potential risks associated with participation were also explained. There were no direct physical or emotional risks; however, participants might feel judged owing to questions regarding work, family, and daily life. It was emphasized that this study was not intended to judge individuals. Additionally, participants were informed that completing the questionnaire might involve a time burden, but it was reiterated that participation was entirely voluntary. While there were no immediate benefits to participation, the study findings could contribute to the improvement of policies and programs to support working households in the future.

This study was approved by the Ethics Committee of Yokohama City University (approval number: 2022-10) and conducted in compliance with relevant ethical guidelines. The data collected during this study, excluding personal information, were shared exclusively with the research team members. Furthermore, all team members regularly participated in ethics training sessions to ensure compliance with ethical research practices and data handling protocols. To ensure the confidentiality of participant data, all collected data were securely stored. Any personally identifiable information was removed, and the data were anonymized. Access to the data was restricted to authorized research personnel only. The data were stored in a secure, password-protected database with appropriate measures in place to prevent data breaches. The data were retained only for the period necessary for the research and were properly disposed of after the study period.

## Results

### Participant characteristics

The primary attributes of the 1,979 respondents are summarized in Table 1.

**Table 1:** Participant characteristics.

Variable	n = 1,979	%	PTPS		P value
			Mean	SD	
**Sex**					
Men	1,039	52.5	24.01	9.52	p < 0.001^1^
Women	940	47.4	25.50	10.06	
**Age (years)**					
20–29	197	10.0	22.71	10.05	p = 0.022^2^
30–39	1,418	72.1	25.01	9.83	
40–49	332	16.9	24.82	9.31	
50–59	19	1.0	24.31	11.02	
**Employment status**					
Employed	1,746	88.9	24.97	9.58	p = 0.056^1^
Unemployed	217	11.1	23.48	10.96	
**Number of children**					
0	368	18.7	19.69	9.01	p < 0.001^2^
1	730	37.0	25.72	9.79	
2	685	34.7	26.04	9.38	
3	157	8.0	26.58	9.65	
≥ 4	32	1.6	24.16	9.87	
**Educational background (n = 1372)**					
High school	119	8.7	23.05	10.46	p = 0.031^2^
Technical school	227	16.5	24.74	9.49	
Bachelor’s degree	781	56.9	25.45	9.50	
Graduate degree	245	17.9	26.02	9.41	
**Household income**					
Less than 6 million yen	362	18.7	24.84	10.06	p = 0.328^2^
6 million to less than 9 million yen	646	33.4	25.39	9.67	
9 million to less than 15 million yen	779	40.3	24.43	9.66	
15 million yen or more	145	7.5	24.71	9.53	

Note: ^1^Welch’s t-test.

^2^Analysis of variance. Employed: includes all respondents who reported having a job, including part-time workers. Unemployed: includes all respondents who reported having no job, including students and homemakers.

The participants had a nearly even sex distribution, with 52.5% men and 47.4% women. Most respondents were in their 30s, comprising 72.1% of the sample. By contrast, only 10.0% of respondents were in their 20s, 16.9% were in their 40s, and a small proportion, 1.0%, were in their 50s. Furthermore, 88.9% of respondents were employed, indicating a high level of workforce participation. In addition, 37.0% of respondents reported having one child, followed closely by 34.7% having two children, whereas 18.7% had no children. Over 80% were engaged in childcare, while more than 70% held a university or graduate degree, and they tended to have high household incomes.

Analysis of the PTPS scores revealed significant differences based on sex, age, educational background, and the number of children. Women had significantly higher PTPS scores (Mean =  25.50, SD =  10.06) than men (Mean =  24.01, SD =  9.52; p <  0.001). Regarding age, participants in their 30s recorded the highest scores (Mean =  25.01, SD =  9.83; p =  0.022). In terms of educational background, a trend was observed whereby higher educational attainment was associated with higher PTPS scores. Participants with a graduate degree reported the highest scores (Mean =  26.02, SD =  9.41), followed by those with a bachelor’s degree (Mean =  25.45, SD =  9.50), showing a significant association (p =  0.031). Additionally, the PTPS scores tended to increase with the number of children. Participants without children had the lowest scores (Mean =  19.69, SD =  9.01), while those with one child had higher scores (Mean =  25.72, SD =  9.79), followed by those with two children (Mean =  26.04, SD =  9.38), and those with three or more children recorded the highest scores (Mean =  26.58, SD =  9.65; p <  0.001). Meanwhile, no significant differences in PTPS scores were observed regarding employment status and household income.

### Distribution and item analysis

The total PTPS score ranged from 6.0 to 42.0, with a mean total score of 24.8 ±  9.68 (mean ±  SD) (Table 2). The average score for the six PTPS items was 4.14 (SD 0.16). The response distribution for each item ranged between 10% and 20%, and the rate of missing responses was between 0.4% and 0.5%. The total scores indicated floor and ceiling effects of 3.88% and 2.70%, respectively. These results suggest that the distribution of PTPS scores is relatively uniform, with minimal response bias. The low rate of missing responses indicates that the survey items were easy to understand and that the burden on respondents was minimal. Additionally, the low percentages of floor and ceiling effects suggest that the scale does not concentrate on extreme values, allowing for an appropriate measurement of a wide range of perceived time poverty levels.

**Table 2:** Item analysis of the PTPS in employed households.

Item	n	Mean	SD	Skewness	Kurtosis	Floor	Ceiling	Missing	G-P, p	I-T, r
I often feel that I do not have time for socializing.	1,971	3.83	1.99	0.07	−1.25	16.43	12.22	0.4	< 0.001	0.555^**^
I often feel that I do not have time for leisure activities.	1,968	4.19	1.98	−0.13	−1.25	11.53	15.34	0.5	< 0.001	0.809^**^
I often feel that I do not have time to travel.	1,968	4.25	2.05	−0.13	−1.32	12.14	19.41	0.5	< 0.001	0.657^**^
I often feel that I do not have time to exercise.	1,967	4.20	2.00	−0.13	−1.25	11.99	16.26	0.6	< 0.001	0.713^**^
I often feel that I do not have time to do the things I like.	1,967	4.30	1.96	−0.21	−1.21	10.42	16.31	0.6	< 0.001	0.837^**^
I often feel that I do not have time to do things that I have planned in my personal life.	1,968	4.07	1.88	−0.07	−1.14	10.67	11.63	0.5	< 0.001	0.795^**^
Total score	1,979	24.8	9.68	−0.2	−0.93	3.88	2.70			

The results of the G-P analysis revealed significant differences (p <  0.001) in all six items between the first and fourth quartiles. The I-T analysis showed correlation coefficients ranging from 0.555 to 0.837 (p <  0.001). These results indicate that all six scale items have sufficient discriminative power and effectively distinguish between groups with high and low perceptions of time poverty. The item-total correlation coefficients ranged from 0.555 to 0.837, suggesting a strong relationship between each item and the overall scale score, which supports the scale’s internal consistency. Meanwhile, the item “I often feel that I do not have time to do the things I like” showed a correlation coefficient of 0.837. Such relatively high correlation coefficients indicate potential redundancy among the items.

### Reliability

Based on Feldt and Duhachek’s standards, the Cronbach’s alpha coefficient for the PTPS was 0.90 (95% CI: 0.89–0.91). In addition, McDonald’s omega coefficient was 0.94, indicating high reliability. Table 3 presents the reliability and inter-item correlation analysis results of the scale items. Cronbach’s alpha coefficients ranged from 0.86 to 0.90, indicating high internal consistency across all items.

**Table 3:** Reliability and inter-item correlation analysis of scale items.

Item	Cronbach’s alpha	Standardized alpha	Lambda	Corrected item-total correlations	Noise ratio	Standard error	Variance of correlations	Median of correlations
I often feel that I do not have time for socializing.	0.90	0.91	0.90	0.66	9.5	0.0043	0.0098	0.63
I often feel that I do not have time for leisure activities.	0.87	0.87	0.85	0.56	6.5	0.0060	0.0126	0.56
I often feel that I do not have time to travel.	0.89	0.89	0.89	0.62	8.2	0.0049	0.0181	0.63
I often feel that I do not have time to exercise.	0.88	0.88	0.88	0.59	7.3	0.0054	0.0195	0.56
I often feel that I do not have time to do the things I like.	0.86	0.86	0.84	0.56	6.2	0.0061	0.0083	0.55
I often feel that I do not have time to do things that I have planned in my personal life.	0.87	0.87	0.86	0.57	6.7	0.0058	0.0058	0.55

Similarly, the standardized alpha coefficients and lambda coefficients exhibited comparable trends. The average inter-item correlations ranged from 0.56 to 0.66, demonstrating moderate to high correlations overall. The noise ratio values ranged from 6.2 to 9.5, showing differences in the explanatory power of variance across items. However, the standard error values, which ranged from 0.0043 to 0.0061, suggest that measurement stability was ensured. Furthermore, the variance and median of the correlations confirmed that the variability among items was adequately maintained.

These results indicate high internal consistency within the scale and that each item is appropriately structured. Meanwhile, the moderate to high inter-item correlations suggest the potential for redundancy among some items. However, considering the noise ratio and correlation variance, each item provides unique information, which supports the overall validity of the scale.

### Exploratory factor analysis

The factor number was examined using a scree plot through parallel analysis (Table 4). Only one factor showed an eigenvalue >  1, whereas all others had values <  1. This suggests that the PTPS has a unidimensional structure. An EFA assuming a one-factor structure was conducted using the EFA dataset (n =  989) with the maximum likelihood method and varimax rotation. All items had sufficient loadings on the common factor, suggesting a consistent factor structure.

**Table 4:** Exploratory factor analysis of the PTPS in employed households (n =  989).

Item	One-factor structure, varimax rotation			Two-factor structure (process), varimax rotation			
	Factor Loadings	Communality	Uniqueness	Factor 1 Loadings	Factor 2 Loadings	Communality	Uniqueness
**Socializing**	0.58	0.34	0.66	0.41	0.41	0.34	0.66
**Leisure**	0.87	0.76	0.24	0.79	0.42	0.81	0.19
**Travel**	0.69	0.48	0.52	0.32	0.72	0.62	0.38
**Exercise**	0.75	0.57	0.43	0.52	0.55	0.57	0.43
**Enjoyable activities**	0.91	0.83	0.17	0.86	0.42	0.91	0.09
**Planned personal activities**	0.85	0.72	0.28	0.59	0.61	0.71	0.29

### Confirmatory factor analysis

A CFA using the CFA dataset (n =  990) was conducted using the maximum likelihood method and varimax rotation (Table 5). The model fit, as indicated by χ²/df, was 0.0527, demonstrating a good fit (p <  0.001). CFI was 0.957, and TLI was 0.929, exceeding the generally accepted threshold of 0.90. The SRMR value was 0.035, indicating a good model fit. The standardized factor loadings ranged from 0.541 to 0.939, demonstrating high factor loadings for all items. Notably, the item “I often feel that I do not have time for things I enjoy” showed the highest standardized loading (0.939). Meanwhile, the RMSEA value was 0.136, with a 90% confidence interval of 0.118 to 0.154. As RMSEA values exceeding 0.10 indicate poor fit, this result suggests that challenges remain regarding the overall model fit.

**Table 5:** Confirmatory factor analysis of the PTPS in employed households (n =  990).

PTPS (α = 0.90/ ω = 0.94)		Standardized loadings
A	**Socializing**	0.541
B	**Leisure**	0.884
C	**Travel**	0.675
D	**Exercise**	0.734
E	**Enjoyable activities**	0.939
F	**Planned personal activities**	0.824
χ²		170.689
df		9
p-value		< 0.001
CFI		0.957
TLI		0.929
RMSEA		0.136
RMSEA 90% CI		0.118–0.154
SRMR		0.035
AIC		21068.044
BIC		21126.657

α, Cronbach’s alpha coefficient; ω, McDonald’s omega coefficient; χ², chi-square; df, degrees of freedom; CFI, comparative fit index; TLI, Tucker–Lewis index; RMSEA, root mean square error of approximation; CI, confidence interval; SRMR, standardized root mean square residual; AIC, Akaike information criterion; BIC, Bayesian information criterion.

### Validity

The correlation analysis between PTPS scores and various daily schedule items showed that sleep duration (mean: 6.65 hours, SD: 1.06, r =  -0.138, p <  0.001) had a significant negative correlation with PTPS scores. This suggests that individuals with higher perceptions of time poverty tend to have shorter sleep durations. Similarly, leisure time (mean: 1.81 hours, SD: 1.62, r =  -0.323, p <  0.001) also showed a significant negative correlation, indicating that those experiencing time poverty tend to have less free time available. Meanwhile, childcare time (mean: 3.83 hours, SD: 3.89, r =  0.195, p <  0.001) exhibited a significant positive correlation with PTPS scores, suggesting that spending more time on childcare is associated with a higher perception of time poverty. By contrast, no statistically significant correlations were observed between PTPS scores and working hours (mean: 7.46 hours, SD: 3.68, r =  -0.022, p =  0.339), commute time (mean: 1.25 hours, SD: 1.28, r =  -0.010, p =  0.671), or housework time (mean: 2.12 hours, SD: 1.66, r =  0.019, p =  0.408). These results suggest that the length of work, commuting, and housework hours might not directly impact the perception of time poverty (Table 6).

**Table 6:** Pearson correlation coefficients between PTPS results and actual time in daily life.

Item	Mean	SD	Skewness	Kurtosis	PTPS	P-value
**Sleep duration**	6.65	1.06	−0.14	4.08	−0.138	p < 0.001
**Working hours**	7.46	3.68	−0.91	3.37	−0.022	p = 0.339
**Commute time**	1.25	1.28	5.10	72.03	−0.010	p = 0.671
**Childcare time**	3.83	3.89	1.47	5.18	0.195	p < 0.001
**Housework time**	2.12	1.66	3.28	533.01	0.019	p = 0.408
**Leisure time**	1.81	1.62	3.39	26.11	−0.323	p < 0.001

The correlation analysis revealed that PTPS scores were significantly negatively correlated with well-being (mean: 7.45, SD: 1.65, r =  -0.215, p <  0.001), suggesting that individuals with a higher perception of time poverty tend to have lower well-being. Similarly, a significant negative correlation was observed with job satisfaction (mean: 6.16, SD: 2.08, r =  -0.210, p <  0.001), indicating that higher time poverty perceptions are associated with lower job satisfaction. Meanwhile, PTPS scores showed a significant positive correlation with psychological distress (mean: 11.10, SD: 5.05, r =  0.181, p <  0.001) and social loneliness (mean: 21.13, SD: 5.62, r =  0.299, p <  0.001). These findings suggest that individuals with higher perceptions of time poverty tend to experience more significant psychological distress and social loneliness (Table 7).

**Table 7:** Pearson correlation coefficients between PTPS results and well-being, psychological distress, social isolation, and job satisfaction.

Item	Mean	SD	Skewness	Kurtosis	PTPS	P-value
**Well-being**	7.45	1.65	−1.00	4.94	−0.215	p < 0.001
**Psychological distress**	11.10	5.05	1.18	4.26	0.181	p < 0.001
**Social loneliness**	21.13	5.62	0.18	2.78	0.299	p < 0.001
**Job satisfaction**	6.16	2.08	−0.67	3.27	−0.210	p < 0.001

## Discussion

This study developed a Japanese version of the PTPS and examined its reliability and validity using data from 1,979 employed households in Yokohama City, collected in the Wave 2 survey of the Hama Study. The findings revealed that certain challenges remain regarding the model fit during the validation process. Nevertheless, the results of this study confirmed that the Japanese version of the PTPS possesses a certain degree of reliability and validity as a tool for assessing perceptions of time poverty. To the best of our knowledge, this study is the first to develop and validate the Japanese version of the PTPS to quantitatively evaluate the concept of subjective time poverty in Japan.

### Reliability and validity of the Japanese version of the perceived time poverty scale

This study confirms that the PTPS demonstrates a certain degree of reliability. The analysis results of the scale were generally consistent with those of the original version, confirming consistent psychometric properties. No response bias was observed across the six PTPS items, and the rate of missing responses remained low (<0.6%). These findings suggest that the survey items were easy to understand for the employed households in Japan targeted in this study, and no issues related to ambiguity or complexity in language were identified.

Furthermore, Cronbach’s alpha coefficients ranged from 0.86 to 0.90, and McDonald’s omega coefficient was 0.94, indicating a very high level of internal consistency for the overall scale. The standardized factor loadings ranged from 0.541 to 0.939, demonstrating high factor loadings for all items. Notably, the item “I often feel that I do not have time for things I enjoy” exhibited the highest standardized loading (0.939), suggesting that this item strongly reflects a key aspect of time poverty. Meanwhile, the item “I often feel that I do not have time to do the things I like” may have a high degree of redundancy among the scale items. However, considering additional information obtained during subsequent analyses and the uniqueness of measuring personal activities, this item was determined to be essential for assessing subjective time poverty. In future longitudinal studies, it will be necessary to continue confirming the sufficient reliability of these items.

The factor analysis results supported the validity of the Japanese version of the PTPS, which confirmed a one-factor structure consistent with the original version of the scale. Although the model fit indexes generally indicated acceptable values, the RMSEA value exceeded 0.1. This suggests that the model fit was insufficient, highlighting areas requiring further improvement.

Regarding individual items, the highest factor loadings were observed for the items “I often feel that I do not have time to do the things I enjoy,” “I often feel that I do not have time for leisure activities,” and “I often feel that I do not have time to do things that I have planned in my personal life.” These items likely reflect the time pressures experienced by employed households, where essential life activities such as work and childcare leave little time for leisure [30]. These findings align with existing literature emphasizing the impact of work–life balance challenges on perceived time availability.

Additionally, items related to socializing and physical activity—such as “I often feel that I do not have time for socializing,” “I often feel that I do not have time to travel,” and “I often feel that I do not have time to exercise”—also showed satisfactory factor loadings. These items may capture broader lifestyle constraints, including social isolation, lack of physical activity, and reduced travel opportunities, which are commonly associated with time poverty among working individuals [31,32]. While these items are particularly relevant in Japan, where long working hours and societal expectations often limit personal time, the challenges identified in the model fit indicate the need for further research.

### Comparison with the original scale and its external validity

Similar to the development of the original scale, this study surveyed a sample spanning various occupational groups and age ranges. The average score across the six PTPS items in the original scale was 3.9 (SD =  1.52), whereas this study yielded a slightly higher mean score of 4.14 (SD =  0.16). This difference may be attributed to the study’s focus on married participants. The results indicated that PTPS scores were significantly higher among individuals in their 30s, those with a university or graduate degree, and those with more children. Given that the survey was conducted in large urban areas, it is possible that households with these characteristics were overrepresented. Meanwhile, the model fit indexes obtained through CFA indicated poorer fit than the original version, with a TLI value of 0.929 and RMSEA value of 0.136. The sample characteristics may influence this.

Regarding the scale items, differences in factor loadings were observed between the original and Japanese versions. Specifically, the factor loadings for “socializing” and “travel” were lower in the Japanese sample, with “socializing” showing a notable difference of 0.58 compared to 0.81 in the original version. This discrepancy may be attributed to the tendency among married and employed households in Japan to prioritize socializing and travel activities less than other commitments, with work and daily life being perceived as central contributors to time poverty. Conversely, such items as “leisure” and “enjoyable activities” showed similarly high factor loadings to those in the original version, confirming that these aspects remain significant components of perceived time poverty in Japan. Although challenges remain regarding model fit, the distribution patterns of the scores generally align with those of the original scale, suggesting that the Japanese version provides a reasonable degree of validity for measuring perceived time poverty within the Japanese cultural context.

### Correlations between perceived time poverty scale results and daily schedule, well-being, depression, social isolation, and job satisfaction

To confirm the construct validity of the PTPS, its relationships with daily schedules and psychosocial variables were examined. A negative correlation was observed between PTPS scores and both sleep and leisure time, suggesting that individuals with higher perceived time poverty tend to experience limitations in discretionary activities such as sleep and leisure [2]. This finding is consistent with previous research indicating that individuals experiencing time poverty struggle to secure sufficient rest, which can adversely affect their mental and physical health. By contrast, no significant correlations were found between PTPS scores and time spent on work, commuting, or household chores. These results suggest that subjective perceptions of time poverty are shaped more by constraints on discretionary time, such as sleep and leisure, rather than by social and family roles themselves. This implies potential tension between individual autonomy and social and family responsibilities [33].

The PTPS showed a negative correlation with overall well-being, suggesting that higher levels of perceived time poverty are associated with lower levels of well-being. This relationship is consistent with previous research findings indicating that individuals who feel a lack of time for personal or leisure activities are more likely to experience stress and dissatisfaction, which can ultimately lead to a decline in their quality of life [34]. In addition, the PTPS showed a positive correlation with social isolation, suggesting that time poverty may limit opportunities for social interaction, particularly among working individuals with limited time for socializing. This relationship is consistent with previous studies indicating that time poverty serves as a barrier to maintaining social connections and participating in community life [27].

A significant negative correlation was observed between PTPS and job satisfaction, suggesting that individuals with higher perceived time poverty tend to have lower job satisfaction. This may be attributed to dissatisfaction stemming from unmet work-related goals and limited career advancement opportunities [1]. Individuals experiencing time poverty often struggle to balance work demands and personal life, which may lead to decreased productivity and job dissatisfaction. This finding aligns with previous studies indicating that time poverty negatively affects job performance and satisfaction [1].

The PTPS revealed significant relationships with various aspects of individuals’ lives, including daily schedules, social connections, and job satisfaction. Quantitatively assessing time poverty could help mitigate its negative impact on individuals and society by informing policies that promote work–life balance, introduce flexible work arrangements, and provide support for household and childcare responsibilities. However, self-reported well-being is highly subjective and may be influenced by such factors as current circumstances and mood, requiring caution in interpreting the results. Similarly, job satisfaction may also be affected by respondents’ current work environment, making it a dynamic factor that requires careful consideration. Future research should incorporate multidimensional scales that can comprehensively evaluate various aspects of well-being and job satisfaction. Additionally, considering multifaceted factors, such as work–life balance and stress levels, in future studies will contribute to a more comprehensive understanding of time poverty and its broader implications.

## Limitations

This study has several limitations. First, test-retest reliability was not assessed, as recommended by the COSMIN guidelines. This limitation arose because of the design constraints of the Hama Study Wave 2, which made it challenging to administer the same questions to participants again. However, building on this longitudinal study, future assessments using the PTPS may provide an opportunity for a more comprehensive evaluation of this aspect. Additionally, the PTPS utilizes a 7-point Likert scale. Since this format may introduce response biases, future studies should consider incorporating a 10-point Visual Analog Scale (VAS) or reverse-coded items.

Second, this study’s geographic scope was limited to Yokohama, Japan. Therefore, further research is needed to generalize the findings to other regions within Japan. However, considering that Yokohama is one of Japan’s most populous and representative designated cities, the findings of this study may offer valuable insights applicable to other regions.

Third, this study targeted employed households in which more than half of the respondents reported high household incomes. This demographic characteristic suggests a potential bias, as the sample represents a relatively socioeconomically stable population. Furthermore, younger single households were not included in the sample, which may limit the generalizability of the findings across different household types. To address these limitations, future research should include a more diverse population and expand the survey to cover a broader range of socioeconomic backgrounds and demographic groups to understand time poverty better.

Despite these limitations, this study is the first to examine the concept of time poverty in Japan and has developed and validated a scale with a certain degree of reliability and validity. By providing a new perspective on quantifying the previously overlooked aspects of time poverty, this study is expected to offer insights that clarify its significance in policy and research contexts.

## Conclusions

The Japanese version of the PTPS has a certain level of reliability and validity. Utilizing this scale to identify households experiencing time poverty is expected to contribute to developing effective support measures and evaluating intervention outcomes. However, challenges remain regarding the model fit, and not all processes recommended by the guidelines for scale development have been fully satisfied. Future research should focus on further validating the scale through longitudinal studies and examining related factors to enhance the comprehensiveness of the assessment.
